# Elevations of neutrophil-to-lymphocyte ratio and C-reactive protein over time as a precursor to anaplastic transformation of papillary thyroid carcinoma: a case report

**DOI:** 10.1186/s40792-024-01991-x

**Published:** 2024-08-19

**Authors:** Masaomi Sen, Ryo Ito, Takeshi Abe, Hiroko Kazusaka, Mami Matsui, Marie Saitou, Ryuta Nagaoka, Tomoo Jikuzono, Iwao Sugitani

**Affiliations:** 1 Department of Endocrine Surgery, Nippon Medical Musashi Kosugi Hospital, 1-383 Kosugi-Cho Nakahara-Ku, Kawasaki-Shi, Kanagawa 211-8533 Japan; 2 Department of Endocrine Surgery, Nippon Medical School Hospital, 1-1-5 Sendagi, Bunkyo-Ku, Tokyo 113-8603 Japan

**Keywords:** Papillary thyroid carcinoma, Anaplastic transformation, Anaplastic thyroid carcinoma, Neutrophil-to-lymphocyte ratio, Biomarker

## Abstract

**Background:**

Papillary thyroid carcinoma rarely undergoes anaplastic transformation. Some risk factors for anaplastic transformation of thyroid cancer are known, but such transformation is difficult to predict in practice. We report a case demonstrating elevations of neutrophil-to-lymphocyte ratio (NLR) and C-reactive protein (CRP) over time as a precursor to anaplastic transformation of thyroid carcinoma.

**Case presentation:**

The patient was an 89 year-old woman with a history of chronic aortic dissection. She was referred to our department after her local doctor detected thyroid nodules. She had previously been found to have multinodular goiter and enlarged left cervical lymph nodes on computed tomography. Her chief complaint was cervical discomfort and hoarseness. Blood tests revealed: white blood cells (WBCs), 4900 /µL; CRP, 0.29 mg/dL; neutrophils, 64.4%; and lymphocytes, 25.4%. A 21 mm mass was identified in the upper left lobe. Left III (16 mm) and left VI (16 mm) lymph node were enlarged on ultrasonography. Fine-needle aspiration cytology diagnosed malignant papillary carcinoma. However, due to the advanced age and medical history of the patient, a non-surgical policy was implemented. The primary tumor grew to 4 cm in diameter by 9 months after diagnosis, and blood tests showed: WBC, 7700 /µL; CRP, 0.18 mg/dL; neutrophils, 65.3%; and lymphocytes, 22.3%. By 10 months after diagnosis, the tumor had increased rapidly in diameter to 8 cm, with blood tests showing: WBC, 6500 /µL; CRP, 1.01 mg/dL; neutrophils, 68.2%; and lymphocytes, 19.3%. Anaplastic transformation of papillary thyroid carcinoma was diagnosed, and the patient was placed on treatment under a policy of best supportive care. Multiple lung metastases appeared 11 months after diagnosis, and blood test results showed: WBC, 13,300 /μL; CRP, 11.28 mg/dL; neutrophils, 93.6%; and lymphocytes, 2.3%. Unfortunately, the patient died of disease progression 63 days after identification of undifferentiated metastasis.

**Conclusions:**

Chances to see the natural history of anaplastic transformation of thyroid cancer are rare. Elevations in NLR and CRP over time may be precursors to anaplastic transformation.

## Background

Anaplastic thyroid carcinoma (ATC) shows an unfortunate course due to rapid disease progression. Clinical manifestations include rapidly enlarging cervical tumor, neck pain, hoarseness, dysphagia, and dyspnea. Imaging findings include invasion of surrounding organs and lymph node metastasis from the neck to the mediastinum. Blood test findings include a mildly elevated white blood cell (WBC) count, elevated C-reactive protein (CRP), and elevated sedimentation rate.

Anaplastic transformation (AT) of thyroid carcinoma is a rare occurrence. Risk factors for AT of thyroid carcinoma include the presence of highly differentiated carcinoma [1], advanced age, and long-term multinodular goiter [2]. In recent years, genes related to the development and progression of thyroid cancer have been identified, and telomerase reverse transcriptase promoter mutations in papillary thyroid carcinoma (PTC) have been identified as a risk factor for AT [3]. However, AT of thyroid cancer in advance remains challenging. We describe herein a case in which the natural history of AT in PTC could be observed and the neutrophil-to-lymphocyte ratio (NLR) and CRP level increased over time.

## Case presentation

**Fig. 1:**
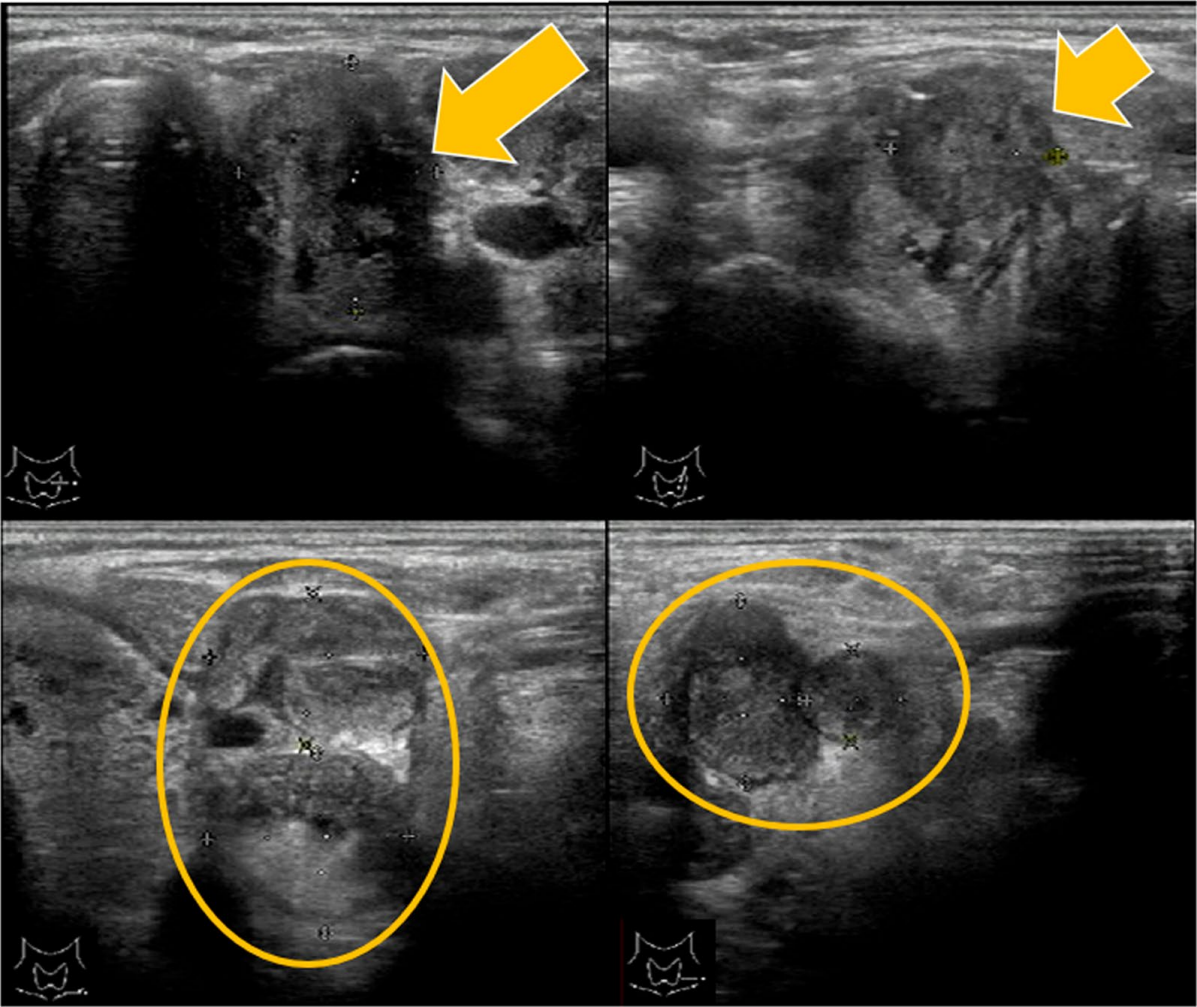
US findings in the neck. There is a 21 × 17 × 13 mm hypoechoic mass in the upper left lobe with an irregular shape and microcalcifications. Invasion of the sternal thyroid muscle is evident, along with enlargement of the left III and left VI lymph nodes

**Fig. 2:**
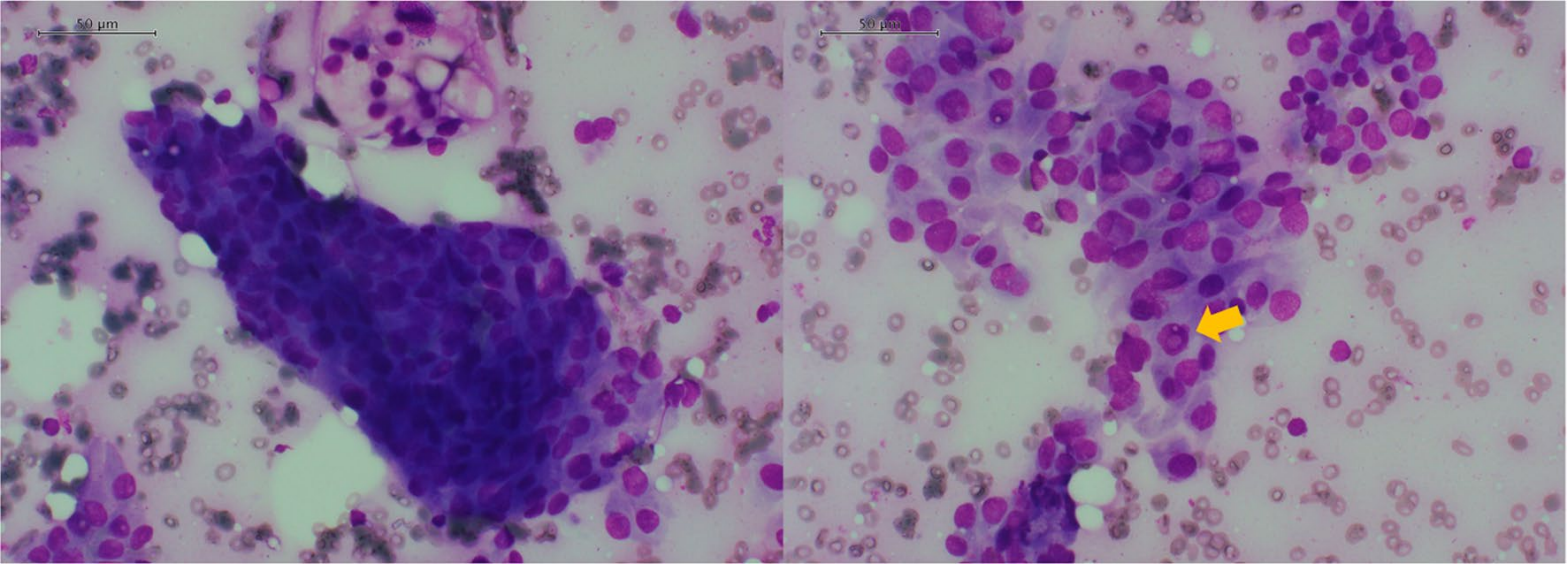
FNAC findings. Left side shows papillary aggregates of follicular cells; right side shows intranuclear cytoplasmic inclusion bodies within follicular cells. Indicated by arrows. Scale bar is 50 μm

**Fig. 3:**
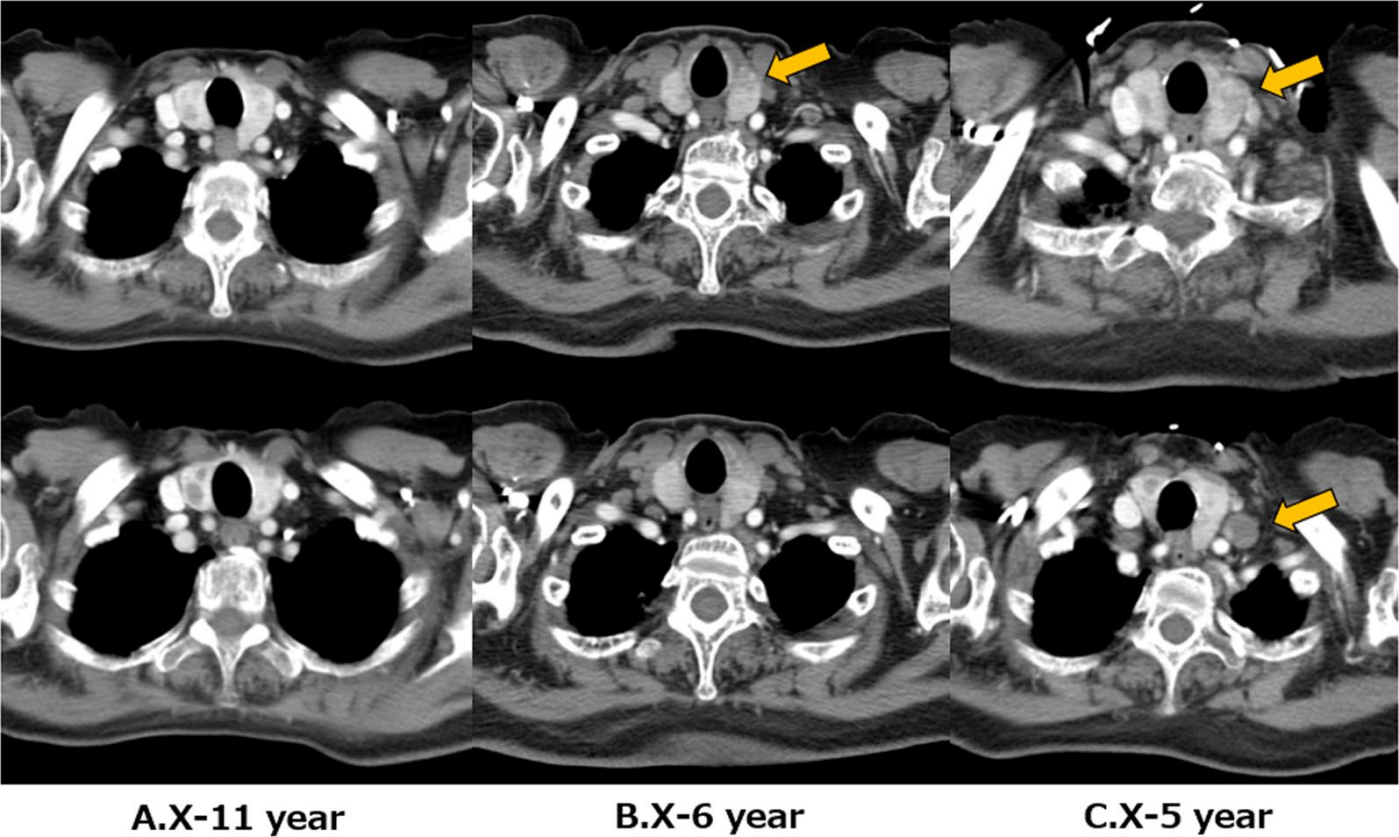
Review of previous CT findings. **A** Multiple thyroid nodules are seen within the thyroid gland. **B** Appearance of primary papillary thyroid carcinoma. **C** Appearance of left VI lymph node metastasis

The patient was an 89 year-old woman who complained of cervical discomfort and hoarseness. She was referred to our department following proximal carotid ultrasound, which revealed a 12 mm nodule in the left lobe of the thyroid gland and an enlarged left lateral cervical lymph node. Her medical history included chronic type B aortic dissection, hypertension, chronic renal failure, myopathy of unknown etiology, low back pain compression fracture, and a postoperative right femoral transverse fracture. Physical examination showed ambulation with a walking aid cart, mild hoarseness, a 2 cm hard nodule on the left side of the neck, and a slightly palpable left cervical lymph node. Blood testing indicated: thyroid stimulating hormone, 6.340 μIU/mL (normal, 0.50–5.00 µIU/mL); free thyroxine, 1.19 ng/mL (normal, 0.90–1.70 ng/dL); free triiodothyronine, 2.74 pg/mL (normal, 2.3–4.0 pg/dL); thyroglobulin, 277.70 ng/mL; anti-thyroglobulin antibodies (TgAb), 13.4 U/mL (normal < 28.0 IU/mL); anti-thyroid peroxidase antibodies (TPOAb), < 9.0 IU/mL (normal < 16.0 IU/mL); WBC count, 4900 /µL; platelet (PLT) count, 232,000 /µL; neutrophils, 64.4%; lymphocytes, 26.4%; monocytes, 6.1%; Blood urea nitrogen, 25.3 mg/dL; creatinine, 1.79 mg/dL; and CRP, 0.28 mg/mL. Ultrasonography (US) revealed a 21 × 17 × 13 mm hypoechoic mass in the upper left lobe with irregular shape and microcalcifications. Invasion of the sternal thyroid muscle was noted, along with enlargement of the left III and left VI lymph nodes (Fig. 1). Fine-needle aspiration cytology (FNAC) showed papillary aggregates of follicular cells and intranuclear cytoplasmic inclusion bodies and confirmed malignancy from the primary tumor and cervical lymph nodes, leading to a diagnosis of PTC (Fig. 2). A review of previous imaging studies revealed multinodular goiter on computed tomography (CT) 11 years earlier (Fig. 3A), papillary carcinoma evident 6 years earlier (Fig. 3B), and metastasis in the left VI lymph node 5 years earlier (Fig. 3C). PTC cT3bN1bM0 was diagnosed, but due to the advanced age of the patient, the history of chronic arterial dissection, and the poor performance status, nonoperative management with close follow-up was chosen.

**Fig. 4:**
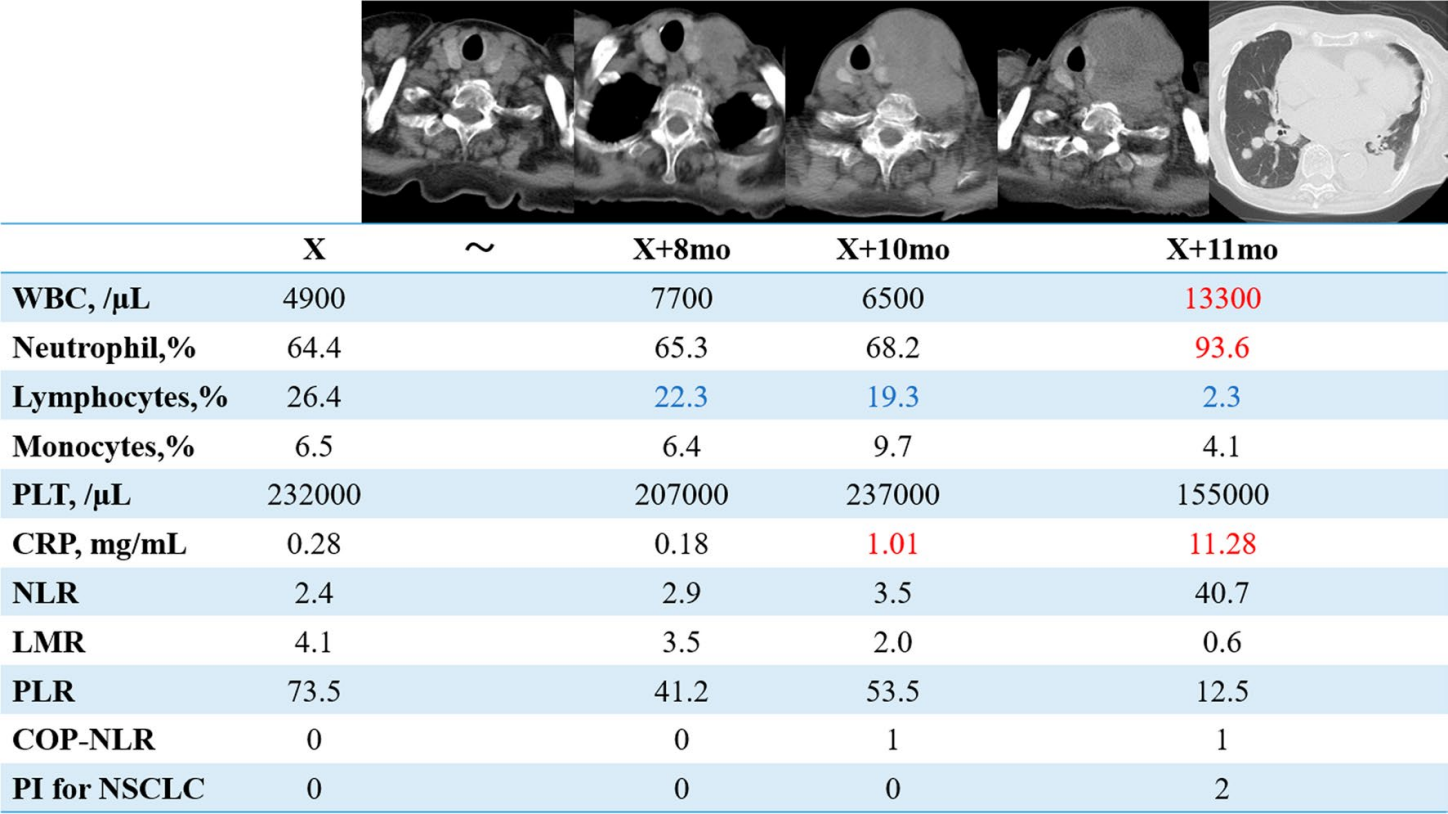
Anaplastic transformation and blood test findings. This figure shows changes in blood tests during the course of anaplastic transformation of papillary thyroid carcinoma. From X + 8 months, tumor growth and decreasing lymphocyte ratio are observed. At X + 10 months, rapid tumor growth, increased NLR, and elevated CRP are observed. At X + 11 months, multiple lung metastases appear, and WBC, NLR, and CRP are all abnormally high

At 3 months post-diagnosis, the patient was diagnosed with acute type A aortic dissection and was conservatively managed in our cardiovascular surgery department, with no progression noted in the PTC. However, subsequent US and CT at 9 months post-diagnosis revealed tumor growth to 46 mm in size, occupying the entire left lobe and extending outward. Blood tests showed: WBC, 7700 /µL; PLT, 207,000 /µL; neutrophils, 65.2%; lymphocytes, 22.3%; monocytes, 6.4%; NLR, 2.9; lymphocyte-to-monocyte ratio (LMR), 4.1; platelet-to-lymphocyte ratio (PLR), 73.5 and CRP, 0.18 mg/mL. At 10 months post-diagnosis, the patient reported neck pain, anorexia, and an enlarged neck mass. CT demonstrated a tumor diameter of 75 × 52 mm and invasion of the esophagus and left internal jugular vein, without distant metastasis. Blood tests revealed: WBC count, 6500 /µL; PLT, 237,000 /µL; neutrophils, 68.2%; lymphocytes, 19.3%; monocytes, 9.7%; NLR, 3.5; LMR,2.0; PLR,53.5 and CRP, 1.01 mg/mL. At 11 months post-diagnosis, tumor diameter increased to 82 × 68 mm and further invaded the cricoid cartilage, with multiple lung metastases and pleural effusion, but no evidence of pneumonia. Blood tests revealed: WBC count, 13,300 /µL; PLT, 155,000 /µL; neutrophils, 93.6%; lymphocytes, 2.3%; monocytes, 4.1%; NLR, 40.7; LMR, 0.6; PLR, 12.4 and CRP, 11.28 mg/mL (Fig. 4). The patient was hospitalized for dysphagia and poor oral intake, receiving fluid and palliative care. Unfortunately, 15 days after admission and 63 days after metastasis, she passed away due to disease progression.

## Discussion

ATC can be suspected based on clinical symptoms, imaging findings, and blood test results.

Leukocytosis and high levels of CRP cases should be considered infectious complications such as cervical abscess and pneumonia. Some ATCs cause tumor necrosis but most cases are associated with extrathyroidal invasion, lymph node metastasis, and distant metastasis. Cervical abscesses often triggered by dental infections or tonsilitis and may spread to the mediastinum. However, this case had no preceding infection and no obvious signs of abscess formation. Also, while large thyroid tumors can cause aspiration pneumonia, there were no signs of pneumonia on the CT when multiple lung metastases and pleural effusion were observed.

The most strongly suspected clinical diagnosis was AT of PTC. Unfortunately, no FNAC or biopsy was performed, so the pathology could not be confirmed.

ATC is known to cause leukocytosis due to a tumor-associated reaction resembling leukocytosis. Leukocytosis is considered a late-stage feature of a specific subtype of ATC that secretes Granulocyte Colony Stimulating Factor or related cytokines, which is a poor prognostic factor for ATC and is included in the Prognostic Index for ATC [4, 5]. Iwasaki et al. reported three cases of AT during lenvatinib treatment for differentiated thyroid cancer and recommended monitoring the clinical course and leukocyte count [6]. The present case was preceded by leukocytosis and increase over time in NLR and CRP levels. Inflammatory markers include NLR, LMR, and PLR, which are prognostic factors for various cancers. NLR is considered useful in differentiating between PTC, poorly differentiated thyroid carcinoma (PDTC), and ATC, with a cutoff of 3.8 between PDTC and ATC [7]. A meta-analysis suggested that pretreatment NLR in thyroid cancer may serve as a biomarker for predicting tumor growth, metastasis, and prognosis [8]. In addition, a group with elevated NLR during the disease course exhibited poorer prognosis compared to a group without elevated NLR [9]. Ahn et al. reported that a low LMR is associated with lower survival in patients with ATC [10]. However, Yamazaki et al. found no significant difference in OS associated with LMR in their study [9, 11]. Furthermore, PLR did not show any association with OS in ATCs [9, 11].

The combination of platelet counts and neutrophil to lymphocyte ratio (COP-NLR) in gastric carcinoma [12], and PI based on WBC and CRP levels in non-small cell lung carcinoma (NSCLC) [13] are used as inflammatory markers. In this case, COP-NLR1 was observed after AT, indicating an increase in NLR without a rise in platelet count, reflecting the elevated NLR. Additionally, PI for NSCLC showed PI 2 at the time of advanced stage with distant metastasis, making prediction of AT challenging. Both markers require further research to confirm their prognostic roles in ATC.

No previous reports have described serial NLRs over time during follow-up in a case resulting in AT. Elevated NLR may help in noticing AT in thyroid cancer.

## Conclusion

Chances to see the natural history of AT of PTC are rare. This case showed elevated NLR and CRP as a precursor to AT.

## Data Availability

The authors declare that all data related to this article are available in this manuscript.

## Abbreviations

ATC: Anaplastic thyroid carcinoma
WBC: White blood cell
AT: Anaplastic transformation
CRP: C-reactive protein
PTC: Papillary thyroid carcinoma
NLR: Neutrophil-to-lymphocyte ratio
TgAb: Anti-thyroglobulin antibodies
TPOAb: Anti-thyroid peroxidase antibodies
PLT: Platelet
US: Ultrasonography
FNAC: Fine-needle aspiration cytology
CT: Computed tomography
LMR: Lymphocyte-to-monocyte ratio
PLR: Platelet-to-lymphocyte ratio
PDTC: Poorly differentiated thyroid carcinoma
COP-NLR: Combination of platelet counts and neutrophil to lymphocyte ratio
NSCLC: Non-small cell lung carcinoma
